# Pervasive RNA Secondary Structure in the Genomes of SARS-CoV-2 and Other Coronaviruses

**DOI:** 10.1128/mBio.01661-20

**Published:** 2020-10-30

**Authors:** P. Simmonds

**Affiliations:** a Nuffield Department of Medicine, University of Oxford, Oxford, United Kingdom; Johns Hopkins Bloomberg School of Public Health

**Keywords:** COVID-19, RNA secondary structure, SARS-CoV-2, persistence

## Abstract

The ultimate outcome of the coronavirus disease 2019 (COVID-19) pandemic is unknown and is dependent on a complex interplay of its pathogenicity, transmissibility, and population immunity. In the current study, severe acute respiratory syndrome coronavirus 2 (SARS-CoV-2) was investigated for the presence of large-scale internal RNA base pairing in its genome. This property, termed genome-scale ordered RNA structure (GORS) has been previously associated with host persistence in other positive-strand RNA viruses, potentially through its shielding effect on viral RNA recognition in the cell. Genomes of SARS-CoV-2 were remarkably structured, with minimum folding energy differences (MFEDs) of 15%, substantially greater than previously examined viruses such as hepatitis C virus (HCV) (MFED of 7 to 9%). High MFED values were shared with all coronavirus genomes analyzed and created by several hundred consecutive energetically favored stem-loops throughout the genome. In contrast to replication-associated RNA structure, GORS was poorly conserved in the positions and identities of base pairing with other sarbecoviruses—even similarly positioned stem-loops in SARS-CoV-2 and SARS-CoV rarely shared homologous pairings, indicative of more rapid evolutionary change in RNA structure than in the underlying coding sequences. Sites predicted to be base paired in SARS-CoV-2 showed less sequence diversity than unpaired sites, suggesting that disruption of RNA structure by mutation imposes a fitness cost on the virus that is potentially restrictive to its longer evolution. Although functionally uncharacterized, GORS in SARS-CoV-2 and other coronaviruses represents important elements in their cellular interactions that may contribute to their persistence and transmissibility.

## Abstract (precis)

The detection and characterization of large-scale RNA secondary structure in the genome of SARS-CoV-2 indicate an extraordinary and unsuspected degree of genome structural organization; this could be effectively visualized through a newly developed contour plotting method that displays positions, structural features, and conservation of RNA secondary structure between related viruses. Such RNA structure imposes a substantial evolutionary cost; paired sites showed greater restriction in diversity and represent a substantial additional constraint in reconstructing its molecular epidemiology. Its biological relevance arises from previously documented associations between possession of structured genomes and persistence, as documented for HCV and several other RNA viruses infecting humans and mammals. Shared properties potentially conferred by large-scale structure in SARS-CoV-2 include increasing evidence for prolonged infections and induced immune dysfunction that prevents development of protective immunity. The findings provide an additional element to cellular interactions that potentially influences the natural history of SARS-CoV-2, its pathogenicity, and its transmission.

## INTRODUCTION

The emergence of severe acute respiratory syndrome coronavirus 2 (SARS-CoV-2) in 2019 in Wuhan, China was the start of a worldwide pandemic of frequently severe, fatal respiratory disease termed coronavirus disease 2019 (COVID-19) (1–4). The ultimate outcome of the pandemic in terms of global morbidity will be devastating with a fear that recurrent episodes of COVID-19 disease will occur regularly unless effective medical interventions such as global immunization can be implemented.

In predicting the future of the COVID-19 pandemic, understanding the ability of a virus to persist at a population level is paramount. Its long-term presence is governed by its intrinsic transmissibility and the ongoing existence of susceptible individuals to maintain transmission. Transmissibility in turn depends on factors such as its route of spread, the resilience of the virus in the environment, and the duration of host immunity after infection and virus clearance. It additionally crucially depends on host persistence; prolonged shedding of infectious virus enables a larger number of susceptible individuals in contact with an infected host to become infected.

In modeling the spread of SARS-CoV-2, information on many of these factors is becoming available. Of greatest concern, populations, such as those in the United Kingdom and the United States which have been severely affected by COVID-19, nevertheless display low levels of population exposure (5–8), indicating that further rounds of infection will not be substantially influenced by herd immunity, even presupposing that infection confers long-term protection. Examples from other respiratory coronaviruses in humans (9–11) or enteric coronaviruses in animals (12–14) do not provide much reassurance on the latter. Furthermore, SARS-CoV-2 is highly transmissible through respiratory routes and close contact (15, 16), it is relatively stable in the environment (17), and SARS-CoV-2 is shed in substantial amounts from respiratory secretions and is infectious through inhalation and ingestion. The final factors, virus persistence with the infected host and the consequent duration of virus shedding, are still incompletely characterized because long-term longitudinal studies of infected individuals are restricted to the few months following the start of the pandemic (see Discussion).

In the current study, the degree of RNA secondary structure within the genomes of SARS-CoV-2 and other human and animal coronaviruses was investigated. This was motivated by our previous observation that human and animal positive-strand RNA viruses capable of virus persistence display a marked, and still largely unexplained, association with their possession of structured RNA genomes (18–20). The nature of the folding of genomic RNA exposed in the cytoplasm during replication differs in many respects from that associated with discrete RNA structures with defined functions, such as replication elements and translation initiation. These typically display highly evolutionarily conserved pairings, often with covariant sites, which create specific structures that interact with viral and cellular RNA sequences and proteins. In contrast, genome-scale ordered RNA structure (GORS) in persistent viruses is distributed throughout the genome and appears agnostic about which specific bases are paired—RNA structures of different hepatitis C virus (HCV) genotypes are quite different from each over most of the genome, yet the overall degree of folding is relatively constant; structure conservation is only apparent within the 3′ end of NS5B and core gene regions and the untranslated genome termini that have known or suspected replication/translation functions (20).

Without structural conservation, GORS can be best detected thermodynamically by comparing the minimum folding energy of a wild-type (WT) sequence with an ensemble of control sequences where the base order of the WT sequence has been shuffled (21, 22). As examples, this sequence order-dependent structure averages at around 8% in HCV, 9% in foot-and-mouth disease virus, and 11% in human pegivirus, similar to the extensively structured rRNA sequences of animals, plants, and prokaryotes (19). The association between possession of GORS and virus persistence in vertebrates extends over all species where information on abilities to persist are documented and has potential predictive value for viruses whose ability to persist is undocumented.

In the current study, we have analyzed genomic sequences of SARS-CoV-2 and members of other coronavirus species and genera infecting humans and other mammals for the presence of GORS. The unexpected and intellectually challenging finding of intense RNA formation in all coronaviruses analyzed has been reviewed in the context of what is currently known about coronavirus persistence in human and other vertebrate hosts.

## RESULTS

### Detection of GORS in coronavirus genomes.

**FIG 1:**
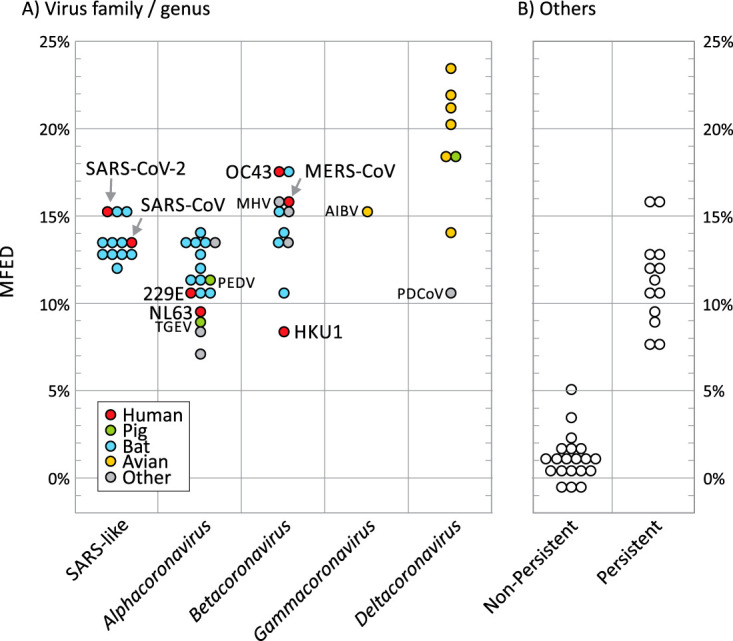
RNA structure prediction in coronaviruses and previously characterized persistent/nonpersistent positive-strand RNA viruses. RNA structure formation was predicted by comparison of minimum folding energies of virus native sequences with those of shuffled controls (MFED value on the *y* axis). (A) Data points represent MFEDs for type member of each currently classified coronavirus species (listed in Table S1 in the supplemental material) and a separate category for SARS-CoV-2, SARS-CoV, and a range of SARS-like viruses infecting bats (sarbecoviruses). Human viruses and widely investigated coronaviruses infecting other species are labeled. AIBV, avian infectious bronchitis virus; MHV, mouse hepatitis virus; PDCoV, porcine deltacoronavirus; PEDV, porcine epidemic diarrhea virus; TGEV, transmissible gastroenteritis virus. (B) MFED values of previously analyzed positive-strand mammalian viruses from a previous study and that reported the association between RNA structure and persistence (19).

A selection of genome sequences of SARS-CoV-2, SARS-CoV, and bat-derived sarbecoviruses were analyzed along with representative members of each classified species of coronavirus (listed in Table S1 in the supplemental material). Quantitation of RNA structure formation in each sequence was based upon comparison of minimum free energy (MFE) on folding the native sequence with those of sequence order shuffled controls (a procedure that maintained mono- and dinucleotide frequencies of the native sequence but otherwise substantially randomized its sequence order). Subtraction of the mean shuffled sequence MFE from the native MFE yielded an MFE difference (MFED) that represents the primary metric for quantifying RNA structure in the current study. SARS-CoV-2, SARS-CoV, and bat-derived homologues all showed evidence for large-scale RNA structure with mean MFED values of around 15% (Fig. 1; raw data listed in Table S1). These values were substantially higher than the MFED values of unstructured viruses (mean value, 1.1%) and indeed of the majority of structured positive-strand RNA viruses displaying host persistence, including HCV (7.5 to 10.7%) and human pegivirus (HPgV) (12.5%) (Fig. 1). However, high MFED values were found in all coronaviruses, particularly in several members of the *Betacoronavirus* genus (range, 8.6 to 17.5%), and extremely high in avian virus members of the genus *Deltacoronavirus* (23.4% in Bulbul coronavirus HKU11-934, the highest recorded in all previous analyses of vertebrate RNA viruses).

10.1128/mBio.01661-20.4 TABLE S1 Representative coronavirus sequences used for RNA structure analysis. Download Table S1, DOCX file, 0.02 MB. Copyright © 2020 Simmonds. 2020 Simmonds This content is distributed under the terms of the Creative Commons Attribution 4.0 International license.

**FIG 2:**
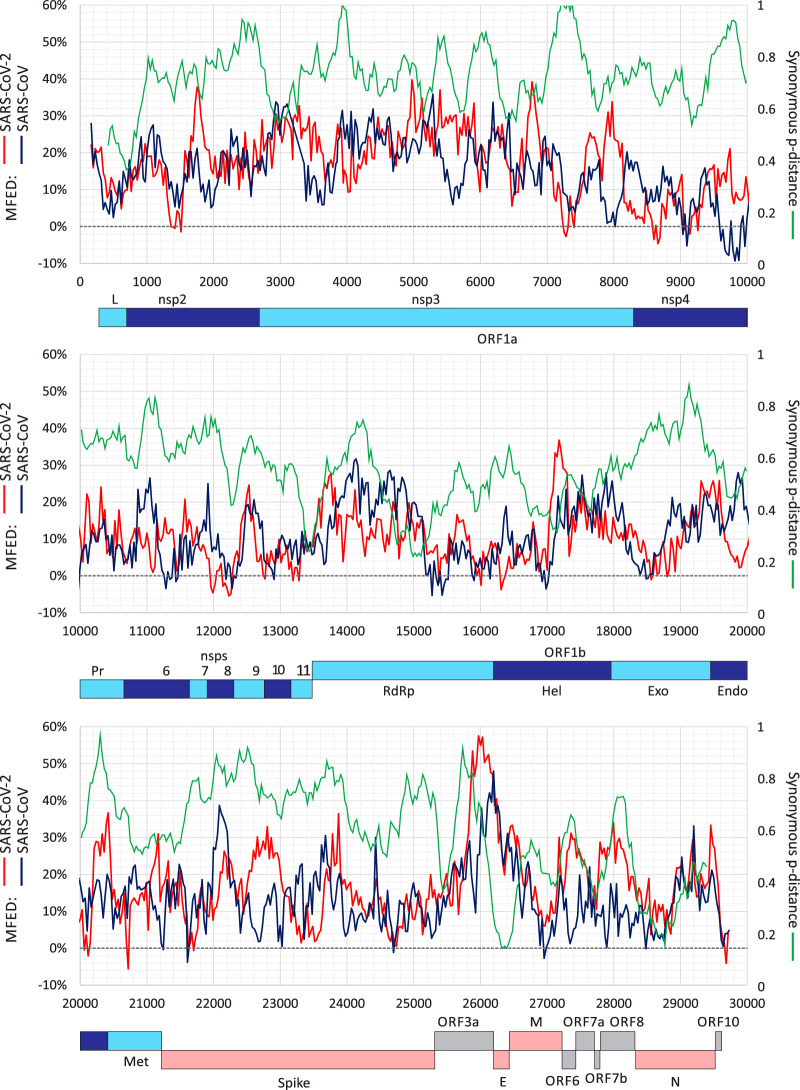
Genome scan of folding energies and synonymous variability. Windowed MFED values of SARS-CoV-2 and SARS-CoV across the genome (left *y* axis) using a fragment size of 350 bases incrementing by 30 bases between fragments. A windowed scan of synonymous p-distances (sequential 300-base fragments incrementing by 30 bases between fragments) of aligned concatenated coding region sequences between SARS-CoV-2 and SARS-CoV is superimposed. A genome diagram of SARS-CoV-2 is drawn to scale under each graph. A listing of the sequences analyzed in provided in Table S3.

By analyzing MFED values for individual sequence fragments used in MFED calculations, it was apparent that SARS-CoV-2 was structured throughout the genome (Fig. 2). Consistently high values of around 20% were found in the nsp2 and nsp3 genes in the ORF1A-encoding region, around 10 to 15% in the remainder of ORF1a and in ORF1b and the spike gene, and a peak of >50% in the ORF3a gene. There was no specific association of elevated MFED values with intergenic regions, the frameshifting site at the ORF1a/OR1b junction or the 5′ or 3′ untranslated regions (UTRs), despite the presence of functional RNA structures in these regions. MFED values in SARS-CoV showed a distribution of elevated values similar to that of SARS-CoV-2 with some differences in parts of nsp3, spike, and ORF3a genes. To investigate the extent to which RNA structure formation imposed constraints on sequence change, variability at synonymous sites in aligned coding sequences of each gene were calculated (green line; Fig. 2). SARS-CoV-2 and SARS-CoV are genetically distinct from each other throughout the genome, but low values indicating constraints did not associate closely with high MFED values or vice versa.

10.1128/mBio.01661-20.5 TABLE S2 Coronavirus sequences used for MFED comparison in different hosts. Download Table S2, DOCX file, 0.02 MB. Copyright © 2020 Simmonds. 2020 Simmonds This content is distributed under the terms of the Creative Commons Attribution 4.0 International license.

10.1128/mBio.01661-20.6 TABLE S3 Coronavirus sequences used for MFED genome scans and contour plots. Download Table S3, DOCX file, 0.02 MB. Copyright © 2020 Simmonds. 2020 Simmonds This content is distributed under the terms of the Creative Commons Attribution 4.0 International license.

**FIG 3:**
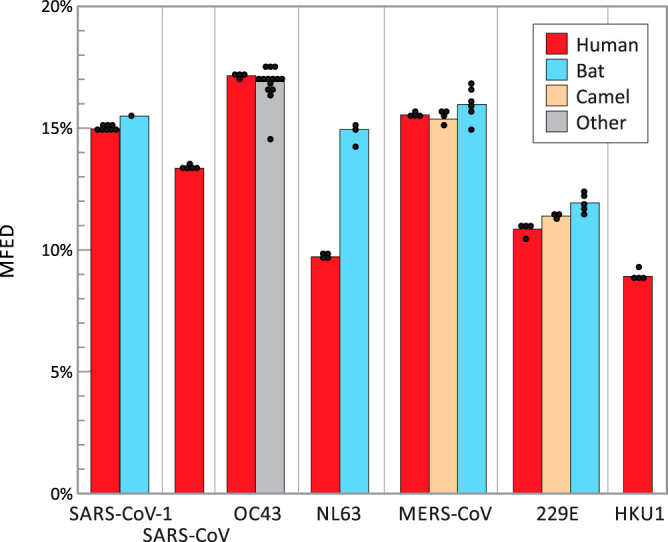
MFED values of human coronaviruses and their closest homologues in other host species. Mean MFED values for selections of representative sequences of each of the seven human coronaviruses and their closest homologues in other mammalian species considered to be their zoonotic source. Sequence selection was limited to up to four for each species listed in Table S2 in the supplemental material and displayed as individual points. Significance tests were not attempted as sequences were phylogenetically related.

Each of the human seasonal coronavirus has a known or suspected zoonotic origin (reviewed in reference 23), with closely related homologues of OC43 identified in cows, NL63, 229E, and Middle East respiratory syndrome CoV (MERS-CoV) in bats. SARS-CoV-2 is closely related to a coronavirus identified in a bat species (2) that may also represent its ultimate zoonotic source. No genetically close homologues of SARS-CoV or HKU1 are known. Each homologue showed a MFED score similar to those of human viruses, although all four bat virus groups were invariably marginally more structured than their human counterparts (SARS-CoV-2, NL63, MERS-CoV, and 229E) (Fig. 3). However, the significance of these differences is difficult to evaluate statistically as the members of each group are phylogenetically related and MFED values derived for individual virus strains do not constitute independent observations.

### Analysis of coronavirus RNA secondary structures.

**FIG 4:**
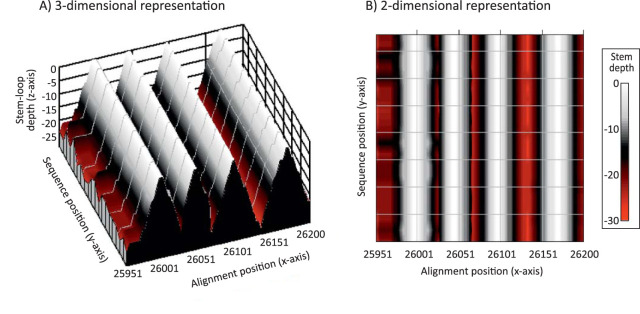
Representation of RNA secondary structure in a region of SARS-CoV-2 as a contour plot. Predicted consensus positions of terminal loops are assigned depths of zero, numbers of sequential pairings in duplex regions plotted on the *z* axis as depths in a 3-dimensional plot (A) and as a color-coded 2-dimensional plot (B). The predicted RNA structure corresponds to a short region of the ORF-3a gene of SARS-CoV-2 analyzed in Fig. 5 to 7.

The genomes of SARS-CoV-2 and other coronaviruses are large, and visualization of their genome-wide RNA structure elements by conventional RNA drawings is problematic. I recently developed a contour plotting method for depicting the positions and variability of secondary structure elements in alignments of virus sequences (20). In this method, pairing predictions from RNAFOLD are recursively scanned for stem-loops and unpaired bases in terminal loops of each are identified and assigned a height of zero on the *z* axis, with genome position and sequence number recorded on the *x* and *y* axes in a 3-dimensional plot (Fig. 4A). Paired bases on either side of the terminal loop were successively plotted according to a color scale that reflects their distance in the stem relative to the terminal loop. The resulting plot therefore provides an approximate visualization of the positions, shapes, and sizes of RNA structure elements across whole alignments. The 3-dimensional representation can be transformed to a 2-dimensional plot with height indicated by color coding (Fig. 4B).

**FIG 5:**
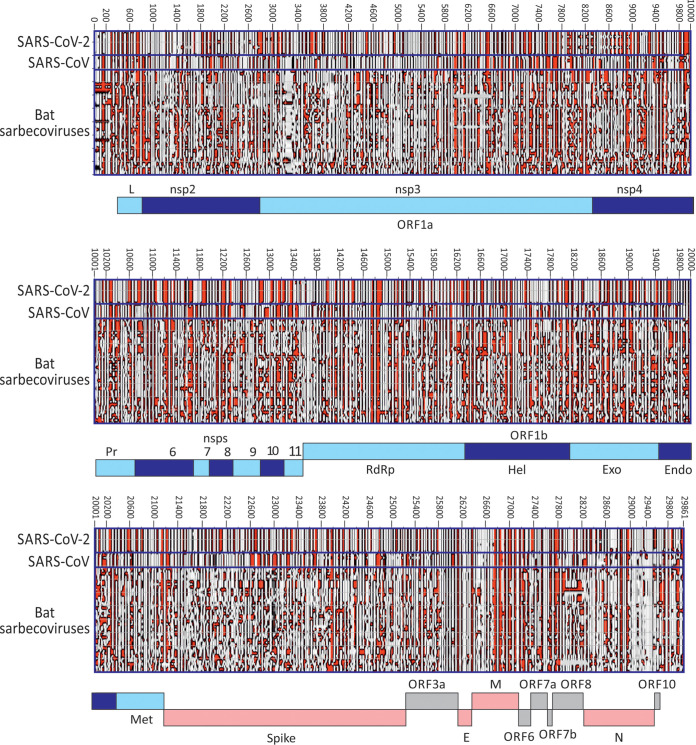
Contour plots of SARS-CoV-2, SARS-CoV, and bat sarbecoviruses. Representation of RNA structure elements in the whole genomes of a selection of SARS-CoV-2 (*n* = 9) and other sarbecoviruses (labeled on *x* axis; listed in Table S3) using the previously described contour plotting method (20).

**FIG 6:**
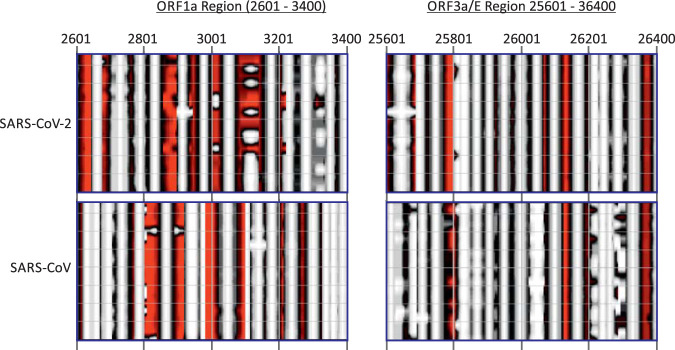
Contour plot comparison of two regions of high MFED values for SARS-CoV-2 and SARS-CoV.

A contour plot was made of an alignment of SARS-CoV-2, SARS-CoV, and bat-derived sarbecoviruses (Fig. 5). SARS-CoV-2 and SARS-CoV variants were minimally divergent, and each produced essentially the same structure predictions. However, these were somewhat different from each other and from bat sarbecoviruses throughout large parts of the genome, highlighting regions with quite different RNA secondary structural organization of duplex and unpaired regions. More focused analyses of two regions of the SARS-CoV-2 and SARS-CoV genomes (positions 2601 to 3400 [in ORF1a] and 25601 to 26400 [in ORF3a/E]) were performed (Fig. 6) to highlight the similarities and differences in base pairings between viruses. Both regions corresponded to areas of high MFED values, 24.5% and 22.32% for SARS-CoV-2 and SARS-CoV in the ORF1a, and 35.8% and 24.7% in ORF3a/E. In the ORF1a region, stem-loop predictions were markedly different between the two viruses despite both viruses showing high MFED values and indeed a consistent pattern of elevation across the entire ORF1a/1b gene, despite these and consistently different actual pairings between the two viruses (Fig. 5).

**FIG 7:**
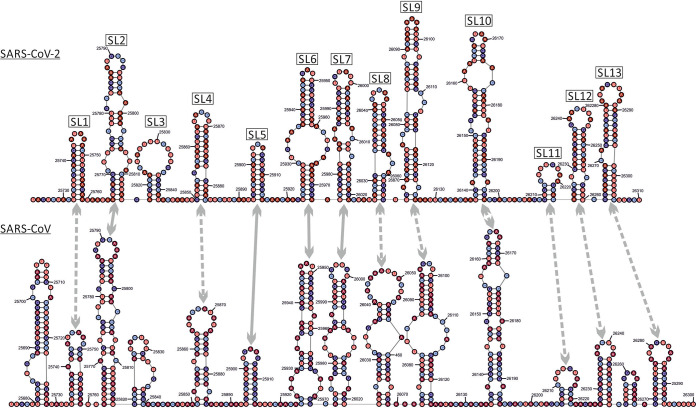
Secondary structure predictions in the ORF3a/E region genomic region of SARS-CoV-2 and SARS-CoV. Drawing of the predicted RNA secondary structure pairings of genome fragments from positions 25601 to 26400 of SARS-CoV-2 and an aligned region of SARS-CoV (24.9% pairwise divergence). Homologous stem-loops between the structure predictions are arrowed. Similar structure and homologous pairings are indicated by a solid line. Similar structures containing nonhomologous pairings are indicated by a dotted line.

In the NS3a/E region, a greater degree of RNA structure conservation was evident in the contour plot. Most predicted stem-loops located to the same places in the alignment, although on closer examination of the base identities of the duplex regions, the actual pairings were nonhomologous in the majority of stem-loops (gray dotted arrows in Fig. 7). Despite alignment of the sequences by nucleotide and amino acid sequence identity (and conservation with other sarbecoviruses), duplexes were often formed by distinct bases in the two viruses. For example, pairings in the first stem-loop in SARS-CoV-2 were displaced 5′ by 2 nucleotide positions in the corresponding SARS-CoV sequence (−2). Pairing displacements of −3 (SL4), −7 (SL8), +3 (SL9), −5 (SL10), +6 (SL12), and −16 (SL13) were observed in otherwise similarly positioned and shaped secondary structure elements, with only SL2 and SL5-SL7 showing evidence for homologous pairing. These observations, recapitulated to even greater extents throughout the remainder of the genome, indicate a considerably faster evolution of RNA secondary structure than their underlying coding sequences. For comparison, RNA structures in OC43 and a set of homologues from animals (pigs, cows, camels, giraffe, deer, and dogs) were visualized in a separate contour plot (see Fig. S1 in the supplemental material). This similarly depicted widely distributed stem-loops through the genome and a degree of structure conservation consistent with the lower degree of sequence divergence between the variants analyzed.

10.1128/mBio.01661-20.1 FIG S1 Contour plot of HCoV-OC43 and homologues in animals. Human OC43 strains (top panel) and a set of homologues from animals (pigs, cows, camels, giraffe, deer, and dogs; bottom panel) were aligned with a genome representation of OC43 strain with GenBank accession no. AY585228, using the annotation provided. Download FIG S1, DOCX file, 1.0 MB. Copyright © 2020 Simmonds. 2020 Simmonds This content is distributed under the terms of the Creative Commons Attribution 4.0 International license.

Secondary structure elements is SARS-CoV-2 and other coronaviruses were primarily comprised of largely unbranched sequential stem-loops. A total of 657 were predicted for SARS-CoV-2, comparable to totals in other coronaviruses (range, 500 to 625), formed from a total of 2,015 duplex regions of 3 or more consecutive base pairs (Table S4). Duplexes in stem-loops were frequently interrupted to avoid paired regions longer than 14 consecutive base pairs. The length distributions of duplex regions were similarly comparable between different coronaviruses (Fig. S2).

10.1128/mBio.01661-20.2 FIG S2 Length distribution and positions of stem-loop duplexes in coronaviruses. (A) Length distribution of uninterrupted duplexes in predicted RNA secondary structures of coronaviruses. (B) Analysis of pairing predictions from the SARS-CoV-2 genome showing the positions and lengths of stem-loop duplexes of length greater than 5 base pairs; the maximum duplex length detected was 14 (*n* = 2). Download FIG S2, DOCX file, 0.3 MB. Copyright © 2020 Simmonds. 2020 Simmonds This content is distributed under the terms of the Creative Commons Attribution 4.0 International license.

10.1128/mBio.01661-20.7 TABLE S4 Predicted RNA structure elements in coronavirus genomes. Download Table S4, DOCX file, 0.02 MB. Copyright © 2020 Simmonds. 2020 Simmonds This content is distributed under the terms of the Creative Commons Attribution 4.0 International license.

### Influence of RNA secondary structure on viral diversity.

While the functional basis for the adoption of pervasive RNA secondary structure is unknown, the apparent requirement for extensive base pairing in SARS-CoV-2 and other coronavirus genomes would be expected to impose constraints on sequence change. Most individual mutations in paired sites would have the effect of weakening RNA secondary structures and lead to a greater phenotypic cost than changes at unpaired sites. For all coronaviruses analyzed, approximately 62 to 67% of bases were predicted be paired (Table S4), and their pairing constraints could therefore lead to a substantial restriction on sequence diversification.

**FIG 8:**
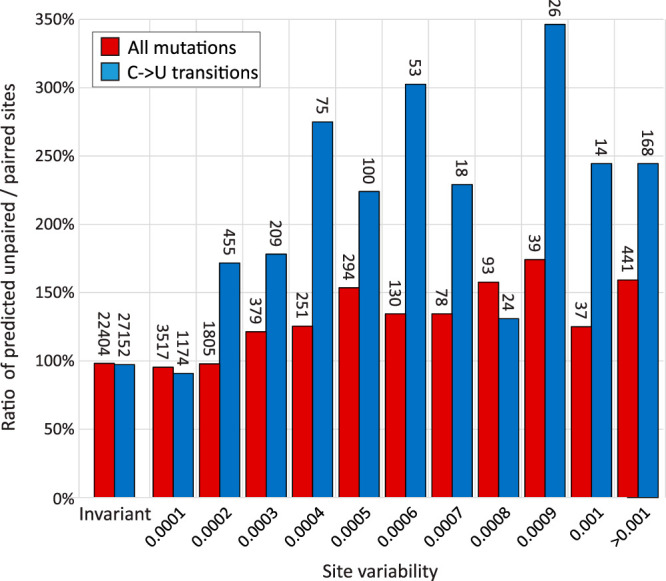
Influence of base pairing on sequence variability. Ratios of unpaired to paired sites predicted by RNAFOLD at invariant sites and sites showing different degrees of site variability. The numbers of sites in each category are shown above the bars. C→U transitions were the most frequent mutations observed in the data set and showed a greater influence of base pairing on their occurrence.

To investigate this, sites in an alignment of 17,518 sequences of SARS-CoV-2 were catalogued for diversity through generating a list of the number of sequence changes at each nucleotide site. The terminal 200 bases at each end of the genome were excluded from the analysis because of lower coverage and greater frequency of sequencing errors in these regions. Overall, a total of 7,064 of the 26,468 nucleotide positions analyzed were polymorphic (27%). Of the variable sites, approximately one half were represented in two or more sequences (sequence divergence ≥ 0.0002), declining steeply thereafter (Fig. S3). Site variability was compared with predictions of whether they were base paired or not base paired using RNAFOLD (Fig. 8). The normalized proportions of unpaired and paired sites were similar for sites showing single mutations (variability, 0.001), but there was increasing overrepresentation of unpaired bases at sites showing greater sequence divergence (nearly twofold for sites with variability greater than 0.008). This overrepresentation was even more marked for C→U transitions (blue bars; up to 3.5-fold overrepresentation). These observations provide evidence for a restricting effect of base pairing on fixation of mutations in the genome.

10.1128/mBio.01661-20.3 FIG S3 Numbers of variable sites in the SARS-CoV-2 genome. Numbers of sites showing different degrees of sequence variability in a total of 17,518 SARS-CoV-2 genomes. Download FIG S3, DOCX file, 0.1 MB. Copyright © 2020 Simmonds. 2020 Simmonds This content is distributed under the terms of the Creative Commons Attribution 4.0 International license.

## DISCUSSION

### Prediction of RNA secondary structure.

The primary evidence for the existence of RNA structure formation in SARS-CoV-2 and other coronavirus genomes was derived from the observation of high MFED values across the genome. Values of 15% in SARS-CoV-2 and 17% in OC43 (and up to 24% in a deltacoronavirus) are unprecedentedly high compared to those documented for HCV (7 to 9%, HPgV (11%) and a range of others reported to possess genome-scale ordered RNA structure (18, 19). MFED calculations identify the sequence order contribution to RNA folding, where elevated values arising from folding energies of native sequences being greater than those of shuffled controls. The use of the NDR shuffling algorithm (24) that preserves these mononucleotide and dinucleotide compositional features, including the unusual underrepresentation of C and overrepresentation of U in most coronavirus sequences (25, 26), provides reassurance that the folding energy differences represent the effects of biologically conditioned sequence ordering to create or maintain RNA secondary structure. Recently published findings of extensive stem-loop formation on physical RNA mapping (27) and elevated MFEs and outlier Z scores (28) that correspond to what are calculated as MFED values in the current study are consistent with conclusions reached about the genome-wide nature of RNA formation.

An independent method to detect and characterize RNA folding, including identifying specific base pairs, is based on the detection of covariance. Covariance-based predictions record compensatory changes in predicted paired bases that maintain binding. In this respect, the extremely limited variability of SARS-CoV-2, SARS-CoV, MERS-CoV, and indeed of each of the sequence data sets of seasonal coronaviruses prevented this approach from being usefully applied in the current study. A second problem is that large-scale RNA structure in other viruses, such as HCV, is not necessarily conserved in the same way as it might be in functional RNA structure elements (20). We recently documented substantial variability in pairing sites both between HCV subtypes in large areas of the genome, with structure conservation restricted to functionally mapped *cis-*acting replication elements in the NS5B region and in stem-loops of undefined function in the core gene (29–34). Covariance detection therefore could be applied to verify pairing sites in HCV, a limitation that potentially extends to other viruses possessing GORS. Evidence for an analogous lack of pairing constraints and comparably rapid evolution of RNA structure is provided by comparison of RNA structure predictions for SARS-CoV-2 and SARS-CoV (Fig. 5 and 7). While there is some similarity in the positions and sizes of predicted stem-loops across their genomes (Fig. 5), particularly apparent in the ORF3a/E region (Fig. 6), the actual pairings forming shared stem-loops were nonhomologous with frequent displacement of paired bases between viruses even though the sizes and spacings of stem-loops were often quite conserved (Fig. 7). This form of “extended” or “inexact” covariance is apparent throughout the SARS-CoV-2 and SARS-CoV genome and supports the idea that it is simple maintenance of pairing rather than functional properties of the stem-loops that are formed that is driving RNA structure formation in coronavirus genomes.

This conclusion is supported by the sheer scale of RNA structure in the SARS-CoV-2 genome. This possesses perhaps 650 or more separate stem-loops throughout coding regions formed through relatively short-range pairing interactions. Predicted pairings were consistent with the distribution of paired and unpaired sites in a recently described SHAPE analysis of the SARS-CoV-2 genome (27). Accepting that many of these predicted structures may derive simply from “overfolding” by energy minimization programs such as RNAFOLD, even half that number would be far too numerous to plausibly possess specific replication functions. Furthermore, areas of high MFED values did not associate with gene boundaries where discrete RNA structure elements may participate in mRNA processing, frameshifting, or other replication functions (35, 36), many elements of which have been recently mapped in the SARS-CoV-2 genome (27, 28, 37). A similar disconnect between MFED values and functional RNA structures in HCV has been described previously (20). As proposed, it appears that it is the folding of RNA, rather than the structures formed, that drive the creation of GORS; how this modifies interactions of the replicating virus with the cell is discussed below.

### Evolutionary constraints of RNA secondary structure.

Notwithstanding the potential inaccuracies of a proportion of specific pairing predictions made by RNAFOLD unassisted by covariance analysis, the marked difference in sequence variability at paired and unpaired sites (Fig. 8) provides evidence that pairing requirements influence SARS-CoV-2 adaptive fitness and potentially limit its longer-term evolutionary trajectory. A striking observation was the frequency-dependent overrepresentation of variability at unpaired sites; sites showing only single sequence mutations were equally well represented predicted paired and unpaired sites, while those showing multiple changes were substantially overrepresented.

The current SARS-CoV-2 data sets are well curated, and consensus sequences generated by next-generation sequencing (NGS) methods, particularly with high read depths, rarely contain sequencing errors. However, even a very low frequency of technical misassignments in a sequence data set of over 17,000 full genome sequences will inevitably contain errors, and these may have contributed to the lack of association with pairing. Nevertheless, a further and potentially more significant contributor to the large number of single sequence mutations (*n* = 3,517) may be the sporadic occurrence of mutations occurring in founder viruses infecting individuals that possess minor fitness defects. These may prevent their propagation and inheritance in other SARS-CoV-2 strains and lack of representation in multiple sequences in the larger data set. The observation that multiply represented and evolutionarily successful mutations were two to three times more likely to occur at unpaired sites indicates that disruption of RNA base pairing imposes a substantial phenotypic penalty on SARS-CoV-2.

Of the 12 possible mutations, C→U transitions were the most commonly observed in the data set, consistent with their previously proposed origin through specific RNA editing events by APOBEC or related cytidine deaminases (25, 38). Transitions induced by C→U changes were more influenced by pairing constraints than other mutations with nearly threefold more occurring at unpaired sites in multiply represented sites. This overrepresentation and their consequent greater likelihood of inheritance or appearing convergently imply a reduced fitness cost that is associated with other mutations. The fact that a substitution of a C for a U at a paired site with G will nevertheless maintain pairing albeit with a lower pairing strength is consistent with this model. The only other mutation that could maintain pairing, A→G, was relatively rare but showed a similar overrepresentation in variable unpaired sites (141%); however, insufficient numbers of mutations occurred for formal frequency analysis (data not shown).

Collectively, the analysis provides evidence that base pairing imposes a substantial constraint on the diversification of SARS-CoV-2 and presumably of other coronaviruses with comparable degrees of RNA structure formation.

### Biological effects of large-scale RNA structure in SARS-CoV-2 and other coronaviruses.

Despite the description of GORS in HCV and a range of other positive-strand RNA viruses, little is known about the biological effects of large-scale RNA structure in viral genomes and how it may influence interactions with the cell. Double-stranded RNA (dsRNA) represents a potent pathogen-associated molecular pattern for a variety of pattern recognition receptors (PRRs) such as RIG-I, MDA5, and oligoadenylate synthetases (OASs 1 to 3) (reviewed in reference 39). Internal base pairing in virus genomes possessing GORS might therefore appear to predispose recognition by PRRs. However, duplexes formed in SARS-CoV-2 and HCV RNA (Fig. 7) (29) are typically interrupted and restricted to consecutive pairing lengths shorter than those recognized by PRRs. Indeed, possession of GORS may have the opposite effect in compacting RNA into forms that may be resistant to binding by PRRs or nucleases. Biophysically, structured genomes take on a globular, compacted appearance on atomic force microscopy, and sequences are inaccessible to external probe hybridization (19), indicating a quite different RNA configuration from unstructured viruses and potentially influencing interactions with the cell. Maintenance of RNA structure is costly in evolutionary terms, since most changes at paired sites, and potentially a proportion at unpaired sites, disrupt RNA folding. In a previous bioinformatic experiment, 5% simulated evolutionary drift of HCV, HPgV, and foot-and-mouth disease virus (FMDV) reduced MFED values of each virus genome by >50% (18). In the real world, longer-term sequence change in these viruses can occur only in a manner that maintains a relatively fixed level of internal base pairing. The observation that SARS-CoV-2 site diversity was substantially influenced by its predicted pairing (Fig. 8) provides a further indication of the potential phenotypic costs of RNA structure disruption.

A further uncertainty about the purpose and mechanisms of GORS-associated structures is the as yet unexplained correlation between RNA structure formation and virus persistence (18, 19). Among many possibilities, we have previously suggested that decreased virus recognition by the innate immune system may fail to activate interferon and other cytokine secretion from infected cells, leading to downstream defects in macrophage and T cell recruitment and maturation. These defects may ultimately blunt adaptive immune responses sufficiently to enable virus persistence. The poor T helper functions were associated with proliferation defects and deletions of reactive CD4 lymphocyte cell responses in those with persistent infections (40–42). Downstream impairment of CD8 cytotoxic T cell and antibody responses may originate from this failure of immune maturation.

On the face of it, the finding that not only SARS-CoV-2, but also all four of the seasonal human coronaviruses possess intensely structured genomes does not square with the previously noted association of GORS with persistence. The human seasonal coronaviruses are considered to cause transient and most often inapparent or mildly symptomatic respiratory infection, notwithstanding the dearth of focused studies on durations of virus shedding and potential sites of replication outside the respiratory tract. Interestingly, repeat testing of individuals with diagnosed NL63, OC43, and 229E infections within 2 to 3 months revealed frequent occurrences of infections with the same virus, >20% in the case of NL63 (9). In many cases, infections were by the same clade of virus and often showed higher viral loads than observed at the original time point. These findings were interpreted as evidence for reinfection as described in previous studies (10, 11), and for some individuals, intermediate samples were obtained and shown to be PCR negative. However, the findings do not rule out persistence over the 3 months of the sampling interval. The observation of NL63 detection in 21% of follow-up samples in a study group where only 1.3% of individuals were initially infected provides some tentative support for the latter possibility. Even if the result of reinfection, the findings demonstrate that seasonal coronaviruses fail to induce any effective form of protective immunity from reinfection even over the short period after primary infection. This resembles findings for HCV, where a potentially comparable immunological defect leads to those who have cleared infection to be readily reinfected with same HCV genotype (43, 44).

In nonhuman hosts, coronavirus infections are typically persistent where investigated. These include bovine coronavirus (BCoV) which establishes long-term, asymptomatic respiratory and enteric infections in cows (45, 46). BCoV is closely related to OC43 in humans and potentially its zoonotic source (23). Although not longitudinally sampled, MERS-CoV was detected at frequencies of >40% in several groups of dromedary camels, similarly indicative of persistence (47) despite its more frequent clearance in infected humans (48). Other coronaviruses showing long-term persistence include mouse hepatitis virus, feline calicivirus (49), and infectious bronchitis virus in birds (50, 51). Pigs are infected with a range of different coronaviruses of variable propensities to establish persistent infections (52–55). Many of the coronaviruses characterized in pigs have arisen in major outbreaks potentially from zoonotic sources, including porcine deltacoronavirus in 2014 from sparrow CoV, and porcine epidemic diarrhea virus in 1971 and swine acute diarrhea syndrome-coronavirus in 2016 from bats (reviewed in reference 56). A lack of host adaptation immediately after recent zoonotic spread may contribute to the various outcomes of pig coronavirus infections. Coronaviruses in bats are distributed in the *Alpha-* and *Betacoronavirus* genera, widespread, highly genetically diverse, and host specific. Establishing whether infections are persistent in bats is problematic in a standard field study setting. However, high detection rates in fecal samples from bats, including 26% and 24% in large samples of Minopterus australis and Minopterus schreibersii in Australia (57), 29% in rhinolophid bats in Japan (58), and 30% in various bat species in the Philippines (59) are strongly indicative of persistence. Overall, coronaviruses clearly have a propensity to persist, although their ability to achieve this may depend on their degree of host adaptation.

Turning to recently emerged coronaviruses in humans, the course of SARS-CoV infections can be prolonged, up to 126 days in fecal samples (60), although little information on persistence was collected before the end of the outbreak. MERS-CoV infections are persistent in camels but show variable outcomes in humans with respiratory detection and fecal excretion typically ceasing 3 to 4 weeks after infection onset (61, 62) but with individual case reports of much longer persistence in some individuals (48). Based on what is known for other coronaviruses, SARS-CoV-2 clearly has the potential for persistence and indeed probably is persistent in its immediate bat source, Rhinolophus affinis (2). Its current presentation as an acute, primarily respiratory infection may represent the typical course of a recently zoonotically transmitted virus with the potential for future adaptive changes to increases its systemic spread and achieve a degree of host persistence apparent in many animal coronaviruses.

Even in the relatively short pandemic period of SARS-CoV-2 6 months after the zoonotic event, relatively long periods of respiratory sample detection and fecal excretion of the virus have been documented, in many cases of greater than 1-month duration (63–67). These occur in both mild and severe cases of COVID-19 in patients, and without comorbidities or evident immune deficits that may separately contribute to persistence. While the world anxiously awaits how SARS-CoV-2 transmissibility and pathogenicity may evolve in future outbreaks, understanding the mechanisms of postzoonotic adaptation of SARS-CoV-2 to humans is of crucial importance. Interactions of SARS-CoV-2 with innate immune pathways potentially modulated by large-scale RNA structure may represent one element in this adaptive process.

## MATERIALS AND METHODS

### SARS-CoV-2 and other coronavirus data sets.

Coronavirus sequences analyzed in the study were downloaded from GenBank and GISAID. A listing of their accession numbers is available from the author upon request.

### RNA structure prediction.

MFED values were calculated by comparing minimum folding energies for WT and sequences shuffled in order by the algorithm NDR. For analysis, coronavirus sequences were split into 350 base sequential sequence fragments incrementing by 15 bases between fragments. For each, MFEs were determined using the RNAFold.exe program in the RNAFold package, version 2.4.2 (68) with default parameters. Summary MFED values (Fig. 1 and 2) were based on mean MFEDs for all fragments in the coding regions of each virus sequence. MFED scans were based on averaging MFEDs from sequence sets for each fragment and plotting values out on the *y* axis, using the midpoint fragment position on the *x* axis (Fig. 3). All shuffling and MFE and MFED determinations were automated in the program MFED scan in the SSE v1.4 package (24) (http://www.virus-evolution.org/Downloads/Software/).

Contour plots were produced using the program StructureDist within the SSE 1.4 package as previously described (20). Briefly, ensemble RNA structure predictions were made from sequential 1,600 base fragments of the alignment incrementing by 400 bases between fragments using the program SubOpt.exe in the RNAFold package. Fragments with pairing predictions consistent in >50% of suboptimal structures were used to construct a consensus contour plot. A listing of paired and unpaired sites was obtained from the Pos.Dat output from StructureDist. Statistics on stem-loop numbers and duplex and terminal loop lengths were obtained from the Stats List.DT1 file generated by the same program.

### Other analyses.

Calculation of synonymous pairwise distances and lists of sequence changes at each site were generated by the programs Sequence Distances, Sequence Changes, and Sequence Join in the SSE package. RNA structure drawings were generated from output from Structure Editor in the RNAstructure package (http://rna.urmc.rochester.edu/RNAstructure.html). Statistical analysis and construction of frequency histograms used SPSS version 26.
