# Supplementary material for: Pervasive RNA Secondary Structure in the Genomes of SARS-CoV-2 and Other Coronaviruses
Source: mBio. 2020 Oct 30;11(6):e01661-20. doi: 10.1128/mBio.01661-20 (PMC7642675; doi:10.1128/mBio.01661-20)
Supplement: TABLE S1 [file mBio.01661-20-st001.docx]

TABLE S1

REPRESENTATIVE CORONAVIRUS SEQUENCES USED FOR RNA STRUCTURE ANALYSIS

| **Group** | **Accession_no** | **Isolate** | **MFED** |
| --- | --- | --- | --- |
| *Sarbecovirus** | MN988713 | Severe acute respiratory syndrome coronavirus 2 | 15.29% |
| *Sarbecovirus** | MN996532 | Bat coronavirus RaTG13 | 15.41% |
| *Sarbecovirus** | FJ882953 | SARS coronavirus MA15 ExoN1 isolate P3pp4 | 13.48% |
| *Sarbecovirus** | KF294457 | SARS-related bat coronavirus isolate Longquan-140 | 12.80% |
| *Sarbecovirus** | KJ473813 | BtRf-BetaCoV/SX2013 | 11.92% |
| *Sarbecovirus** | KP886809 | Bat SARS-like coronavirus YNLF_34C | 13.23% |
| *Sarbecovirus** | KJ473814 | BtRs-BetaCoV/HuB2013 | 13.68% |
| *Sarbecovirus** | MG772934 | Bat SARS-like coronavirus isolate bat-SL-CoVZXC21 | 15.51% |
| *Sarbecovirus** | KY352407 | Severe acute respiratory syndrome-related coronavirus | 12.77% |
| *Sarbecovirus** | GU190215 | Bat coronavirus BM48-31/BGR/2008 | 12.75% |
| *Sarbecovirus** | JX993988 | Bat coronavirus Cp/Yunnan2011 | 13.57% |
| *Sarbecovirus** | MK211374 | Coronavirus BtRl-BetaCoV/SC2018 | 12.82% |
|  |  |  |  |
| *Alphacoronavirus* | KF430219 | Bat coronavirus CDPHE15/USA/2006 | 13.35% |
| *Alphacoronavirus* | JQ989270 | Rousettus bat coronavirus HKU10 isolate 183A | 11.40% |
| *Alphacoronavirus* | KJ473807 | BtRf-AlphaCoV/HuB2013 | 11.42% |
| *Alphacoronavirus* | AF304460 | Human coronavirus 229E | 10.37% |
| *Alphacoronavirus* | KF294380 | Lucheng Rn rat coronavirus isolate Lucheng-19 | 13.35% |
| *Alphacoronavirus* | LC119077 | Ferret coronavirus FRCoV4370 | 7.35% |
| *Alphacoronavirus* | HM245925 | Mink coronavirus strain WD1127 | 8.14% |
| *Alphacoronavirus* | EU420138 | Miniopterus bat coronavirus 1 | 13.37% |
| *Alphacoronavirus* | EU420139 | Bat coronavirus HKU8 strain AFCD77 | 13.89% |
| *Alphacoronavirus* | KJ473806 | BtMr-AlphaCoV/SAX2011 | 13.06% |
| *Alphacoronavirus* | KJ473809 | BtNv-AlphaCoV/SC2013 | 10.86% |
| *Alphacoronavirus* | AF353511 | Porcine epidemic diarrhea virus strain CV777 | 11.14% |
| *Alphacoronavirus* | DQ648858 | Bat coronavirus (BtCoV/512/2005) | 11.87% |
| *Alphacoronavirus* | EF203064 | Bat coronavirus HKU2 strain HKU2/GD/430/2006 | 10.63% |
| *Alphacoronavirus* | AY567487 | Human Coronavirus NL63 | 9.65% |
| *Alphacoronavirus* | KY073745 | NL63-related bat coronavirus strain BtKYNL63-9b | 13.73% |
| *Alphacoronavirus* | AJ271965 | Transmissible gastroenteritis virus | 8.75% |
|  |  |  |  |
| *Betacoronavirus* | AY585228 | Human coronavirus OC43 strain ATCC VR-759 | 17.57% |
| *Betacoronavirus* | KM349742 | Betacoronavirus HKU24 strain HKU24-R05005I | 15.22% |
| *Betacoronavirus* | AY597011 | Human coronavirus HKU1 genotype A | 8.60% |
| *Betacoronavirus* | AY700211 | Murine hepatitis virus strain A59 | 15.55% |
| *Betacoronavirus* | KF636752 | Bat Hp-betacoronavirus/Zhejiang2013 | 15.26% |
| *Betacoronavirus* | KC545383 | Betacoronavirus Erinaceus/VMC/DEU/2012 | 13.60% |
| *Betacoronavirus* | EF065509 | Bat coronavirus HKU5-1 | 13.50% |
| *Betacoronavirus* | EF065505 | Bat coronavirus HKU4-1 | 17.50% |
| *Betacoronavirus* | KU762338 | Rousettus bat coronavirus isolate GCCDC1 356 | 10.86% |
| *Betacoronavirus* | EF065513 | Bat coronavirus HKU9-1 | 14.13% |
|  |  |  |  |
| *Gammacoronavirus* | IBACGB | Avian infectious bronchitis virus | 14.98% |
|  |  |  |  |
| *Deltacoronavirus* | JQ065048 | Wigeon coronavirus HKU20 strain HKU20-9243 | 14.21% |
| *Deltacoronavirus* | FJ376619 | Bulbul coronavirus HKU11-934 | 23.45% |
| *Deltacoronavirus* | JQ065043 | Porcine coronavirus HKU15 strain HKU15-155 | 18.30% |
| *Deltacoronavirus* | FJ376622 | Munia coronavirus HKU13-3514 | 20.24% |
| *Deltacoronavirus* | JQ065044 | White-eye coronavirus HKU16 strain HKU16-6847 | 21.92% |
| *Deltacoronavirus* | JQ065047 | Night-heron coronavirus HKU19 strain HKU19-6918 | 18.50% |
| *Deltacoronavirus* | JQ065049 | Common-moorhen coronavirus HKU21 strain HKU21-8295 | 21.19% |
| *Deltacoronavirus* | EU111742 | Coronavirus SW1 | 10.71% |
|  |  |  |  |
