# Supplementary material for: Pervasive RNA Secondary Structure in the Genomes of SARS-CoV-2 and Other Coronaviruses
Source: mBio. 2020 Oct 30;11(6):e01661-20. doi: 10.1128/mBio.01661-20 (PMC7642675; doi:10.1128/mBio.01661-20)
Supplement: TABLE S2 [file mBio.01661-20-st002.docx]

TABLE S2

CORONAVIRUS SEQUENCES USED FOR MFED COMPARISON IN DIFFERENT HOSTS

| **Run_name** | **Sequence** | **Host** | **MFED** |
| --- | --- | --- | --- |
| SARS-CoV-2 | MN988713 | Human | 15.05% |
| SARS-CoV-2 | MT093571 | Human | 14.85% |
| SARS-CoV-2 | MT049951 | Human | 14.98% |
| SARS-CoV-2 | MT039890 | Human | 14.99% |
| SARS-CoV-2 | MT027064 | Human | 14.92% |
| SARS-CoV-2 | MT007544 | Human | 15.04% |
| SARS-CoV-2 | MN994467 | Human | 15.10% |
| SARS-CoV-2 | MN996528 | Human | 15.01% |
| SARS-CoV-2 | MN996527 | Human | 14.99% |
| Sarbecovirus* | MN996532 | Bat | 15.07% |
| SARS-CoV-1 | FJ882953 | Human | 13.33% |
| SARS-CoV-1 | AY654624 | Human | 13.33% |
| SARS-CoV-1 | FJ882926 | Human | 13.45% |
| SARS-CoV-1 | FJ882943 | Human | 13.27% |
| SARS-CoV-1 | HQ890531 | Human | 13.35% |
| NL63_H | AY567487 | Human | 9.59% |
| NL63_H | KY674916 | Human | 9.81% |
| NL63_H | JQ765564 | Human | 9.78% |
| NL63_H | MG428700 | Human | 9.73% |
| NL63_B | KY073744 | Bat | 15.14% |
| NL63_B | NC_048216 | Bat | 14.22% |
| NL63_B | KY073746 | Bat | 14.97% |
| HKU1_H | AY597011 | Human | 8.91% |
| HKU1_H | KF686342 | Human | 8.85% |
| HKU1_H | DQ415899 | Human | 9.28% |
| HKU1_H | KY674921 | Human | 8.75% |
| OC43_H | AY585228 | Human | 17.19% |
| OC43_H | KY369907 | Human | 17.03% |
| OC43_H | KF530088 | Human | 17.15% |
| OC43_H | KF530060 | Human | 17.23% |
| OC43_OM | KU558922 | Bovine | 16.63% |
| OC43_OM | MG757140 | Bovine | 16.57% |
| OC43_OM | KY419105 | Pig | 16.33% |
| OC43_OM | EF424622 | Camel | 17.45% |
| OC43_OM | EF424623 | Camel | 17.57% |
| OC43_OM | EF424624 | Camel | 17.53% |
| OC43_OM | FJ425186 | Deer | 17.08% |
| OC43_OM | MG518518 | Deer | 16.96% |
| OC43_OM | MH810163 | Deer | 14.53% |
| OC43_OM | KF906249 | Camel | 17.02% |
| OC43_OM | MN514964 | Camel | 17.05% |
| OC43_OM | MN514962 | Camel | 16.96% |
| OC43_OM | JX860640 | Dog | 16.93% |
| OC43_OM | KX432213 | Dog | 16.75% |
| 229E_H | AF304460 | Human | 10.44% |
| 229E_H | KY967357 | Human | 11.04% |
| 229E_H | KY996417 | Human | 10.90% |
| 229E_H | JX503060 | Human | 10.93% |
| 229E_C | KT368905 | Camel | 11.49% |
| 229E_C | MF593473 | Camel | 11.43% |
| 229E_C | JQ410000 | Camel | 11.34% |
| 229E_B | KT253272 | Bat | 11.50% |
| 229E_B | KY073747 | Bat | 11.91% |
| 229E_B | KT253269 | Bat | 11.64% |
| 229E_B | KY073748 | Bat | 12.16% |
| 229E_B | MN611517 | Bat | 12.38% |
| MERS-CoV_H | KC164505 | human | 15.57% |
| MERS-CoV_H | KT026454 | human | 15.59% |
| MERS-CoV_H | KT156561 | human | 15.49% |
| MERS-CoV_H | KM027255 | human | 15.55% |
| MERS_CoV_C | MH734115 | Camel | 15.67% |
| MERS_CoV_C | MF598699 | Camel | 15.18% |
| MERS_CoV_C | MF598619 | Camel | 15.73% |
| MERS_CoV_C | MG923479 | Camel | 15.41% |
| MERS_CoV_B | MF593268 | Bat | 16.89% |
| MERS_CoV_B | KC869678 | Bat | 16.50% |
| MERS_CoV_B | NC_034440 | Bat | 16.08% |
| MERS_CoV_B | MG021451 | Bat | 14.91% |
| MERS_CoV_B | MG596802 | Bat | 15.89% |
| MERS_CoV_B | MG596803 | Bat | 15.75% |
