# Supplementary material for: Pervasive RNA Secondary Structure in the Genomes of SARS-CoV-2 and Other Coronaviruses
Source: mBio. 2020 Oct 30;11(6):e01661-20. doi: 10.1128/mBio.01661-20 (PMC7642675; doi:10.1128/mBio.01661-20)
Supplement: TABLE S3 [file mBio.01661-20-st003.docx]

TABLE S3

CORONAVIRUS SEQUENCES USED FOR MFED GENOME SCANS AND CONTOUR PLOTS

| **Accession_no** | **Group** | **Isolate** |
| --- | --- | --- |
| MN988713 | SARS-CoV-2 | Severe acute respiratory syndrome coronavirus 2 isolate |
| MT093571 | SARS-CoV-2 | Severe acute respiratory syndrome coronavirus 2 isolate |
| MT049951 | SARS-CoV-2 | Severe acute respiratory syndrome coronavirus 2 isolate |
| MT039890 | SARS-CoV-2 | Severe acute respiratory syndrome coronavirus 2 isolate SNU01 |
| MT027064 | SARS-CoV-2 | Severe acute respiratory syndrome coronavirus 2 isolate |
| MT007544 | SARS-CoV-2 | Severe acute respiratory syndrome coronavirus 2 isolate |
| MN994467 | SARS-CoV-2 | Severe acute respiratory syndrome coronavirus 2 isolate |
| MN996528 | SARS-CoV-2 | Severe acute respiratory syndrome coronavirus 2 isolate WIV04 |
| MN996527 | SARS-CoV-2 | Severe acute respiratory syndrome coronavirus 2 isolate WIV02 |
| FJ882953 | SARS-CoV-1 | SARS coronavirus MA15 ExoN1 isolate P3pp4 |
| AY654624 | SARS-CoV-1 | SARS coronavirus TJF |
| FJ882926 | SARS-CoV-1 | SARS coronavirus ExoN1 |
| FJ882943 | SARS-CoV-1 | SARS coronavirus MA15 ExoN1 |
| HQ890531 | SARS-CoV-1 | SARS coronavirus MA15 ExoN1 isolate d4ym1 |
| KF294457 | Bat sarbecovirus* | SARS-related bat coronavirus isolate Longquan-140 |
| GQ153543 | Bat sarbecovirus* | Bat SARS coronavirus HKU3-8 |
| GQ153547 | Bat sarbecovirus* | Bat SARS coronavirus HKU3-12 |
| DQ084200 | Bat sarbecovirus* | bat SARS coronavirus HKU3-3 |
| KJ473813 | Bat sarbecovirus* | BtRf-BetaCoV/SX2013 |
| KY770860 | Bat sarbecovirus* | Bat coronavirus isolate Jiyuan-84 |
| KJ473812 | Bat sarbecovirus* | BtRf-BetaCoV/HeB2013 |
| KJ473811 | Bat sarbecovirus* | BtRf-BetaCoV/JL2012 |
| KU182964 | Bat sarbecovirus* | Bat coronavirus isolate JTMC15 |
| KY938558 | Bat sarbecovirus* | Bat coronavirus strain 16BO133 |
| DQ648856 | Bat sarbecovirus* | Bat coronavirus (BtCoV/273/2005) |
| DQ412042 | Bat sarbecovirus* | Bat SARS coronavirus Rf1 |
| JX993987 | Bat sarbecovirus* | Bat coronavirus Rp/Shaanxi2011 |
| KP886809 | Bat sarbecovirus* | Bat SARS-like coronavirus YNLF_34C |
| DQ071615 | Bat sarbecovirus* | Bat SARS coronavirus Rp3 |
| KY417143 | Bat sarbecovirus* | Bat SARS-like coronavirus isolate Rs4081 |
| MK211377 | Bat sarbecovirus* | Coronavirus BtRs-BetaCoV/YN2018C |
| KY770858 | Bat sarbecovirus* | Bat coronavirus isolate Anlong-103 |
| KJ473816 | Bat sarbecovirus* | BtRs-BetaCoV/YN2013 |
| KY417145 | Bat sarbecovirus* | Bat SARS-like coronavirus isolate Rf4092 |
| FJ588686 | Bat sarbecovirus* | Bat SARS CoV Rs672/2006 |
| KY417142 | Bat sarbecovirus* | Bat SARS-like coronavirus isolate As6526 |
| MK211375 | Bat sarbecovirus* | Coronavirus BtRs-BetaCoV/YN2018A |
| KY417147 | Bat sarbecovirus* | Bat SARS-like coronavirus isolate Rs4237 |
| KY417148 | Bat sarbecovirus* | Bat SARS-like coronavirus isolate Rs4247 |
| KJ473815 | Bat sarbecovirus* | BtRs-BetaCoV/GX2013 |
| KY417146 | Bat sarbecovirus* | Bat SARS-like coronavirus isolate Rs4231 |
| KC881006 | Bat sarbecovirus* | Bat SARS-like coronavirus Rs3367 |
| KC881005 | Bat sarbecovirus* | Bat SARS-like coronavirus RsSHC014 |
| KJ473814 | Bat sarbecovirus* | BtRs-BetaCoV/HuB2013 |
| DQ648857 | Bat sarbecovirus* | Bat coronavirus (BtCoV/279/2005) |
| DQ412043 | Bat sarbecovirus* | Bat SARS coronavirus Rm1 |
| MG772934 | Bat sarbecovirus* | Bat SARS-like coronavirus isolate bat-SL-CoVZXC21 |
| MG772933 | Bat sarbecovirus* | Bat SARS-like coronavirus isolate bat-SL-CoVZC45 |
| KY352407 | Bat sarbecovirus* | Severe acute respiratory syndrome-related coronavirus strain |
| GU190215 | Bat sarbecovirus* | Bat coronavirus BM48-31/BGR/2008 |
| JX993988 | Bat sarbecovirus* | Bat coronavirus Cp/Yunnan2011 |
| KF569996 | Bat sarbecovirus* | Rhinolophus affinis coronavirus isolate LYRa11 |
| MK211374 | Bat sarbecovirus* | Coronavirus BtRl-BetaCoV/SC2018 |

*Used for contour plots only
