# Supplementary material for: Pervasive RNA Secondary Structure in the Genomes of SARS-CoV-2 and Other Coronaviruses
Source: mBio. 2020 Oct 30;11(6):e01661-20. doi: 10.1128/mBio.01661-20 (PMC7642675; doi:10.1128/mBio.01661-20)
Supplement: FIG S1 [file mBio.01661-20-sf001.docx]

SUPPLEMENTARY FIGURE 1 – CONTOUR PLOT OF HCoV-OC43 AND HOMOLOGUES IN ANIMALS


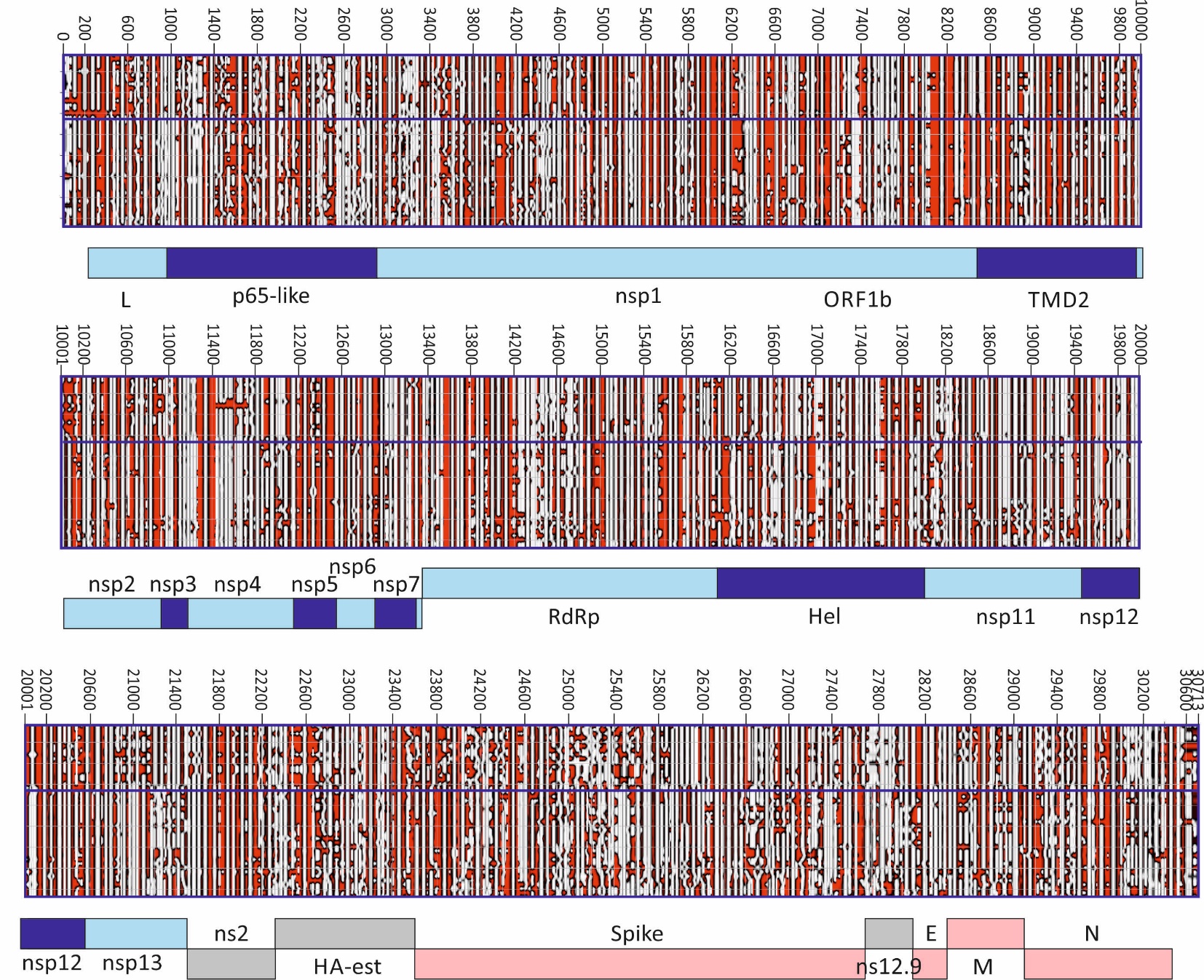


Human OC43 strains (upper panel) and a set of homologues from animals (pigs, cows, camels, giraffe, deer and dogs; lower panel) were aligned with a genome representation of OC43 strain AY585228, using the annotation provided.
