# Supplementary material for: Pervasive RNA Secondary Structure in the Genomes of SARS-CoV-2 and Other Coronaviruses
Source: mBio. 2020 Oct 30;11(6):e01661-20. doi: 10.1128/mBio.01661-20 (PMC7642675; doi:10.1128/mBio.01661-20)
Supplement: FIG S2 [file mBio.01661-20-sf002.docx]

FIGURE S2

LENGTH DISTRIBUTION AND POSITIONS OF STEM-LOOP DUPLEXES IN CORONAVIRUSES


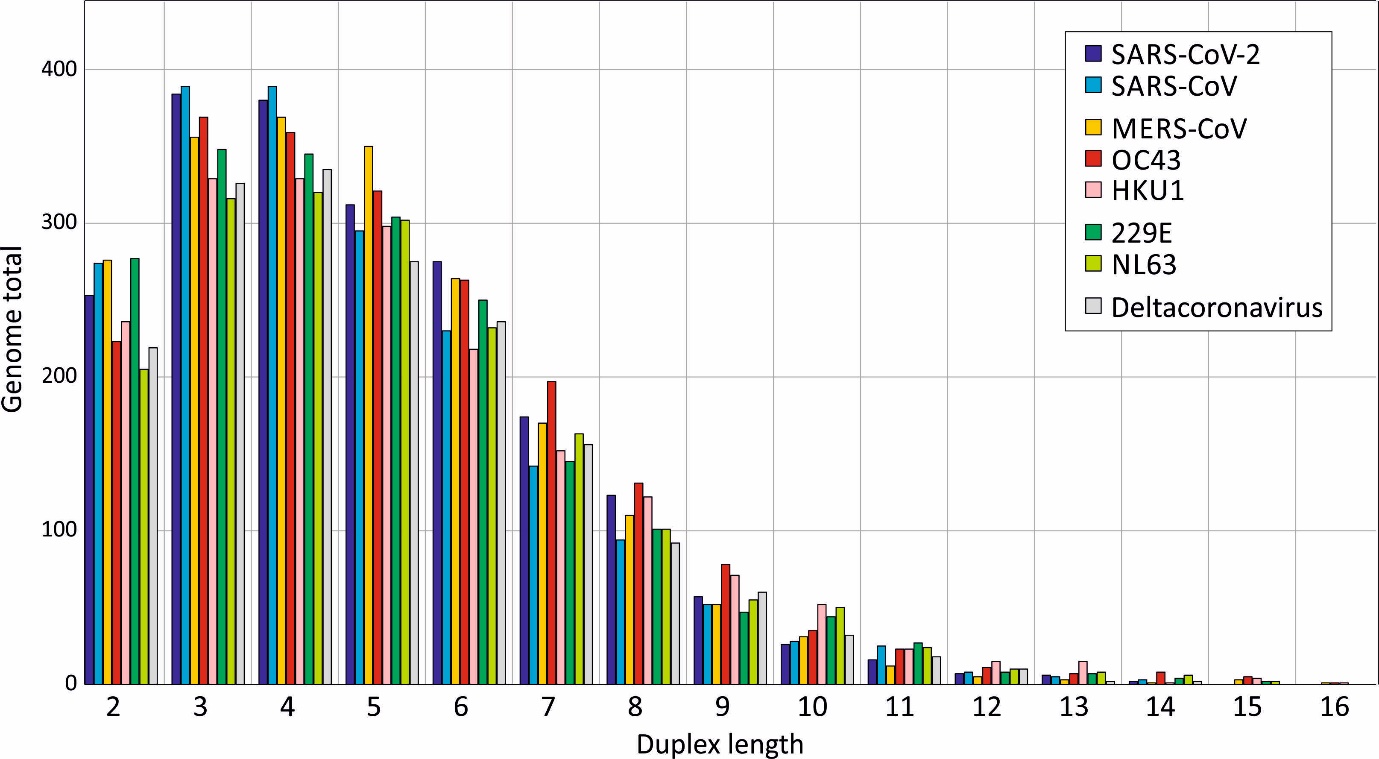
A)


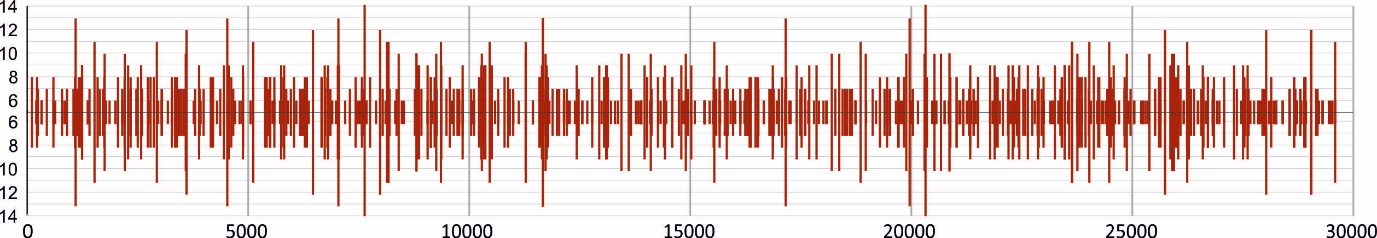
B)

A) Length distribution of uninterrupted duplexes in predicted RNA secondary structures of coronaviruses. (B) Analysis of pairing predictions from the SARS-CoV-2 genome showing the positions and lengths of stem-loop duplexes of length greater than 5 base pairs; the maximum duplex length detected was 14 (n=2).
