# Supplementary material for: Pervasive RNA Secondary Structure in the Genomes of SARS-CoV-2 and Other Coronaviruses
Source: mBio. 2020 Oct 30;11(6):e01661-20. doi: 10.1128/mBio.01661-20 (PMC7642675; doi:10.1128/mBio.01661-20)
Supplement: TABLE S4 [file mBio.01661-20-st004.docx]

TABLE S4

PREDICTED RNA STRUCTURE ELEMENTS IN CORONAVIRUS GENOMES

| **Virus** | | | | | | | **Duplexes** | **Max. length** | **Stem-loops** | **Length range** | **Prop. paired** |
| --- | --- | --- | --- | --- | --- | --- | --- | --- | --- | --- | --- |
| SARS-CoV-2 | | | | | | | 2015 | 14 | 657 | 3-44 | 63.0% |
| SARS-CoV | | | | | | | 1934 | 14 | 645 | 3-39 | 65.7% |
| MERS-CoV | | | | | | | 2003 | 16 | 627 | 3-18 | 62.3% |
| OC43 | | | | | | | 2034 | 18 | 616 | 3-24 | 66.9% |
| HKU1 | | | | | | | 1877 | 16 | 557 | 3-21 | 65.7% |
| 229E | | | | | | | 1909 | 15 | 473 | 3-16 | 66.5% |
| NL63 | | | | | | | 1798 | 17 | 500 | 3-18 | 65.6% |
| Deltacoronavirus | | | | | | | 1763 | 14 | 547 | 3-18 | 64.2% |
|  |  |  |  |  |  |  |  |  |  |  |  |
