# Supplementary material for: Pervasive RNA Secondary Structure in the Genomes of SARS-CoV-2 and Other Coronaviruses
Source: mBio. 2020 Oct 30;11(6):e01661-20. doi: 10.1128/mBio.01661-20 (PMC7642675; doi:10.1128/mBio.01661-20)
Supplement: FIG S3 [file mBio.01661-20-sf003.docx]

FIGURE S3

NUMBERS OF VARIABLE SITES IN THE SARS-CoV-2 GENOME


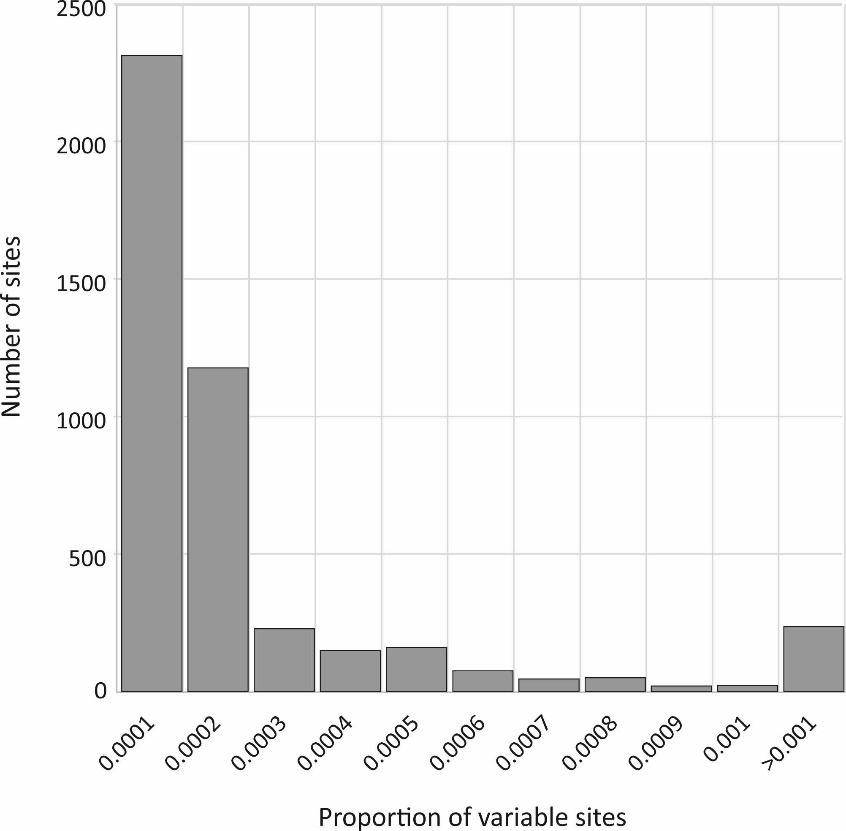


Numbers of sites showing different degrees of sequence variability in a total of 17518 SARS-CoV-2 genomes.
